# Clinical impact of chronic obstructive pulmonary disease on non-cystic fibrosis bronchiectasis. A study on 1,790 patients from the Spanish Bronchiectasis Historical Registry

**DOI:** 10.1371/journal.pone.0177931

**Published:** 2017-05-18

**Authors:** David De la Rosa, Miguel-Angel Martínez-Garcia, Rosa Maria Giron, Montserrat Vendrell, Casilda Olveira, Luis Borderias, Luis Maiz, Antoni Torres, Eva Martinez-Moragon, Olga Rajas, Francisco Casas, Rosa Cordovilla, Javier de Gracia

**Affiliations:** 1 Department of Pneumology, Hospital Plató, Barcelona, Spain; 2 Department of Pneumology, Hospital de Requena, Valencia, Spain; 3 Departament of Pneumology, Instituto de Investigación Sanitaria, Hospital Universitario de la Princesa, Madrid, Spain; 4 Department of Pneumology, Hospital Josep Trueta Biomedical Research Institute (IDIBGI), Girona, Spain; 5 Department of Pneumology, Hospital Regional Universitario de Málaga, Instituto de Biomedicina de Málaga (IBIMA), Facultad de Medicina de Málaga, Spain; 6 Department of Pneumology, Hospital General San Jorge, Huesca, Spain; 7 Department of Pneumology, Hospital Ramón y Cajal, Madrid, Spain; 8 Respiratory Institute, Hospital Clinic i Provincial, Barcelona, Spain; 9 Department of Pneumology, Hospital de Sagunto, Valencia, Spain; 10 Department of Pneumology, Hospital Universitario San Cecilio, Granada, Spain; 11 Department of Pneumology, Hospital Universitario de Salamanca, Salamanca, Spain; 12 Department of Pneumology, Hospital Universitari Vall d’Hebron, Barcelona, Spain; Lee Kong Chian School of Medicine, SINGAPORE

## Abstract

**Background:**

Few studies have evaluated the coexistence of bronchiectasis (BE) and chronic obstructive pulmonary disease (COPD) in series of patients diagnosed primarily with BE. The aim of this study was to analyse the characteristics of patients with BE associated with COPD included in the Spanish Bronchiectasis Historical Registry and compare them to the remaining patients with non-cystic fibrosis BE.

**Methods:**

We conducted a multicentre observational study of historical cohorts, analysing the characteristics of 1,790 patients who had been included in the registry between 2002 and 2011. Of these, 158 (8.8%) were registered as BE related to COPD and were compared to the remaining patients with BE of other aetiologies.

**Results:**

Patients with COPD were mostly male, older, had a poorer respiratory function and more frequent exacerbations. There were no differences in the proportion of patients with chronic bronchial colonisation or in the isolated microorganisms. A significantly larger proportion of patients with COPD received treatment with bronchodilators, inhaled steroids and intravenous antibiotics, but there was no difference in the use of long term oral or inhaled antibiotherapy. During a follow-up period of 3.36 years, the overall proportion of deaths was 13.8%. When compared to the remaining aetiologies, patients with BE associated with COPD presented the highest mortality rate. The multivariate analysis showed that the diagnosis of COPD in a patient with BE as a primary diagnosis increased the risk of death by 1.77.

**Conclusion:**

Patients with BE related to COPD have the same microbiological characteristics as patients with BE due to other aetiologies. They receive treatment with long term oral and inhaled antibiotics aimed at controlling chronic bronchial colonisation, even though the current COPD treatment guidelines do not envisage this type of therapy. These patients’ mortality is notably higher than that of remaining patients with non-cystic fibrosis BE.

## Introduction

Commercial development and scientific research related to bronchiectasis (BE) were scarce throughout most of the 20th century due to the low prevalence of this disease as a consequence of continuous health improvements, to such an extent that it became an “orphan disease” [1]. We are now going through a phase in which this disease is receiving great attention as a result of better diagnosis via thoracic high-resolution computed tomography (HRCT), increased scientific evidence on the effect of drugs such as macrolides or inhaled antibiotics and the establishment of new BE aetiologies [2]. There has been some controversy in recent years, however, as regards whether chronic obstructive pulmonary disease (COPD) [3] should be recognised as a BE aetiology, given the absence of any evidence proving a causal relationship, although studies have increasingly shown a high prevalence of BE in COPD patients, particularly those with more advanced stages of the latter disease [4,5]. The coexistence of both diseases causes more symptoms, an accelerated functional decline, further complications and increased mortality [6–9]. However, most data come from series of patients with COPD and there are few studies that evaluate the coexistence of both diseases in series of patients diagnosed primarily with BE.

The creation of BE registries is helping us obtain epidemiological and clinical data about this disease, including its relationship with COPD. In population-based registries, information is collected from all those centres where patients with an initial diagnosis of BE are diagnosed and/or treated, which makes it possible to estimate the scale of the disease in the corresponding geographical areas [10]. This overview facilitates the control of selection biases when conducting epidemiological studies. The Spanish Bronchiectasis Historical Registry (SBHR) was founded in 2002 by the Spanish Society for Pneumology and Thoracic Surgery (SEPAR). Its aim was to collate a large number of BE patients to further the understanding of the disease and identify its most common causes and clinical characteristics, as well as the most commonly isolated microorganisms and the most commonly followed treatment regimens.

The aim of this study is to analyse the characteristics of the patients diagnosed with BE related to COPD who are included in the SBHR and compare them to the remaining patients with non-cystic fibrosis (CF) BE.

## Material and methods

We conducted a multicentre observational study of historical cohorts by analysing adult patients included in the SBHR between June 2002 and November 2011 who had been diagnosed with BE in various specialised outpatient clinics throughout Spain.

### Structure of the registry

The SBHR was an electronic database, accessible through the SEPAR website (www.separ.es/bronquiectasias), to which all affiliated pulmonologists were invited to contribute. The inclusion criteria were a primary diagnosis of BE, obtained via thoracic CT, bronchography or, exceptionally, clinical-radiological criteria. Data were entered into an electronic form divided into five sections: identification/clinical data, aetiology, bronchial colonisation, lung function and current treatment regimen. Each patient was examined by the SBHR coordination team before being validated. In case of omissions or inconsistencies, applicable corrections were requested from the corresponding researcher as a quality-control measure.

### Study population

In this study, we analysed every patient included in the SBHR with non-CF BE. We rejected those with CF, since this group shows very specific clinical and evolutive characteristics. The cases in which COPD was considered a BE-associated disease were compared to the remaining cases.

### Registry variables

The SBHR collected several clinical data: age, gender, height/weight, year in which symptoms started, year in which the BE was diagnosed, smoking history, presence and type of expectoration (white, white-yellow or green), presence of haemoptysis and/or sinusitis, exacerbations and mortality. An exacerbation was defined as an acute and sustained presentation of changes in the characteristics of sputum (increased volume, consistency, purulence or haemoptysis), and / or increased dyspnoea not due to other causes [11]. Information about the BE diagnosis method was requested: CT, HRCT, bronchography or clinical-radiological criteria. The BE extension was also recorded as localised, bilateral or diffuse (if it affected 4 lobes or more).

Researchers were asked to include the data of the spirometry performed closest to the moment of inclusion in the registry, with the patient in a stable condition. As recommended in the SEPAR guidelines for performing spirometry, post-bronchodilator values were used in cases where this test was performed. The lung function data requested were: absolute values of forced vital capacity (FVC) and maximum expiratory volume in one second (FEV_1_) in l/s, FEV_1_/FVC ratio and oxygen saturation (SatO_2_). Subsequently, the theoretical values of FVC and FEV_1_ were calculated through the formulas of Roca et al [12].

Researchers had to assign an aetiology to the BE of each patient. To ensure that the data were as homogeneous as possible, researchers were urged to follow specific recommendations proposed by SEPAR concerning the aetiological diagnosis. These recommendations were later included in the SEPAR 2008 Bronchiectasis Diagnosis and Treatment Guidelines [11]. In cases where BE was considered to have an unknown aetiology, a record of the diagnostic tests used to rule out specific BE causes was requested [11]. It was left to each researcher’s discretion to assign COPD or asthma as the cause of BE in a given patient, but a recommendation was made to reasonably exclude any other possible aetiology. The COPD diagnosis was based on the GOLD Guidelines in force since 2002 [13]. If a diagnosis of COPD-related BE was established in a patient without obstructive pattern in the spirometry incorporated in the registry, the coordinating team contacted the physician responsible for the diagnosis to confirm that spirometries had ratified the obstruction at some point of his clinical evolution and thereby supported the diagnosis. Otherwise, the patient was not considered to have COPD and was transferred to the idiopathic BE group. Thus, COPD-related BE was established only if the diagnosing physician thought, in addition to the confirmation of spirometric obstruction, that COPD was a key factor due to smoking and medical history, or due to the presence of emphysema in CT (data not recorded in the registry). Moreover, COPD was excluded as a BE-associated disease in many smokers with an obstructive spirometric pattern, if COPD was not considered by the researcher to be responsible for the BE or clinical symptoms, and if these patients presented some other condition that clearly influenced their clinical evolution and could therefore be considered the main cause of BE.

All patients with three or more positive cultures for the same microorganism during a period of 6 months, in different samples taken at intervals of one month, were considered to have chronic bronchial colonisation (CBC) [14]. The researcher had to classify each patient as either colonised or not colonised at the point of inclusion in the registry.

Furthermore, the current treatment regimen at the time of patient inclusion in the registry was requested: oral antibiotic therapy (in exacerbations, cyclic or continuous), inhaled antibiotic therapy (cyclic or continuous), intravenous antibiotic therapy, bronchodilator treatment (BD), inhaled corticosteroids (ICS) or surgical treatment.

### Ethics statement

Researchers guaranteed patients’ confidentiality, in accordance with the data protection law [15] by making their identification data anonymous and by using alphanumeric codes. Information analysis was always aggregated, never individual. Every researcher agreed to comply with the Guidelines to Good Clinical Practice.

### Statistical analysis

We conducted a descriptive analysis with central tendency and dispersion measures for quantitative variables (mean and standard deviation) and frequency distribution for qualitative variables. The T-Student test (or the ANOVA test in those cases with three or more categories) was used to compare independent quantitative variables; the Chi-square test was used for independent categorical variables.

In order to assess whether the presence of COPD in patients with BE was associated with increased mortality, a survival analysis was conducted using the Cox regression with the follow up data available in the SBHR. This analysis was adjusted for those variables which, according to researchers, were clinically relevant, such as age, gender, body mass index, CBC by PA, FEV_1_ (% predicted), smoking habit (active, previous smoker or always non-smoker) and BE extension (localised, bilateral or diffuse). The survival analysis was graphically represented by the Kaplan Meier curves and the log-rank test was used to compare the curves.

## Results

**Fig 1:**
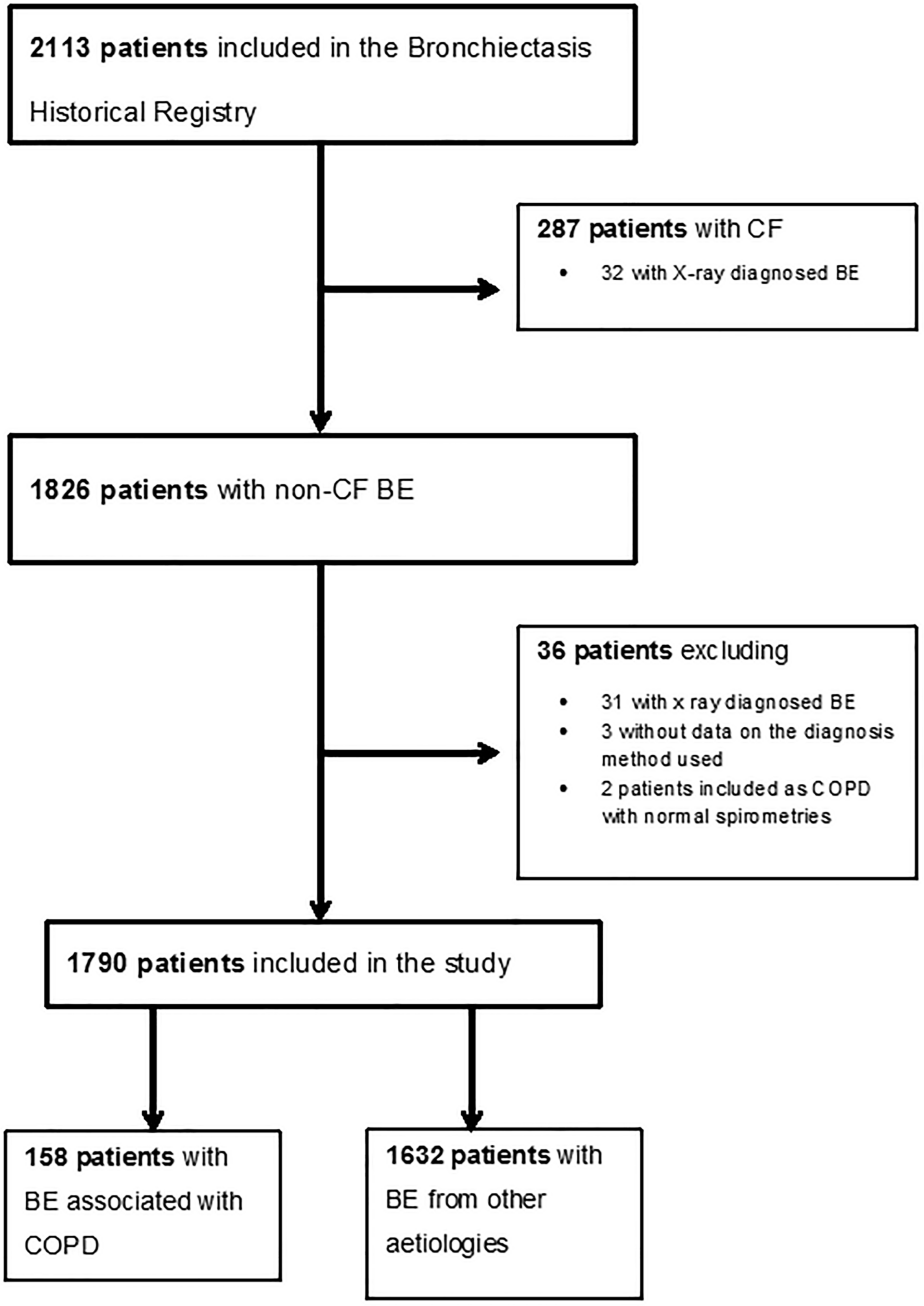
Study flowchart. BE: bronchiectasis; CF: cystic fibrosis; COPD: chronic obstructive pulmonary disease.

Between June 2002 and November 2011, 2,113 patients from 36 health centres in 11 different autonomous communities were included prospectively in the SBHR. 287 of these (13.6%) had CF BE and were excluded from the current study. We have also excluded 36 more patients due to incomplete or incorrect data (Fig 1). We have, therefore, analysed the remaining 1,790 patients with non-CF BE. 158 of these (8.8%) were registered as having BE related to COPD. The remaining registered aetiologies were: unknown (27.7%), post-tuberculous (21.2%), non-tuberculous infection (13%), primary immunodeficiencies (10.7%), asthma (6.1%), ciliary disease (3.3%) and up to 21 further aetiologies. A complete list of individual and grouped aetiologies can be seen in S1 File.

### General, radiological and clinical data

There were differences in the BE diagnostic method between non-COPD patients and COPD patients (p = 0.026). The methods used were HRCT (84.8% vs. 77.2% of the cases, respectively), conventional CT (14.4% vs. 22.2%) and bronchography (0.8% vs. 0.6%). As shown in Table 1, there was a significantly higher percentage of men in the COPD group: they were older at the time of inclusion in the registry, at the onset of symptoms and at the time BE was diagnosed, and the period between the onset of symptoms and the diagnosis of BE was shorter. In these patients, distribution of BE was more frequently bilateral and a greater proportion had daily expectoration, which was most frequently white. There was also a higher proportion of patients with annual exacerbations (72% vs. 48%; p = 0.02).

**Table 1:** General, clinical and radiological data.

	BE without COPD (n = 1,632)	BE with COPD (n = 158)	*p*
**Male sex** (% of patients)	40.8	89.9	**< 0.001**
**General data** (mean ± sd)			
**Age** (years)	63.7 ± 18.6	76.4 ± 10.4	**< 0.001**
**Age at onset of symptoms** (years)	35.6 ± 23.9	56.5 ± 16.9	**< 0.001**
**Age at BE diagnosis** (years)	48.9 ± 20.9	65.7 ± 10.9	**< 0.001**
**Diagnosis delay** (years between onset of symptoms and diagnosis of BE)	13.4 ± 16.2	9.2 ± 11.5	**0.002**
**Clinical data** (% of patients)			
**Current or previous smoking**	32.6	96.8	**< 0.001**
**Daily expectoration**	50.8	65.2	**0.001**
**Type of expectoration**			
**• White** **• White-yellow** **• Yellow or green**	**•** 31.4 **•** 32.7 **•** 19.0	**•** 40.5 **•** 36.7 **•** 15.2	**0.004**
**Sinusitis**	26.5	7.2	**< 0.001**
**Haemoptysis**	33.3	27.2	**ns**
**Presence of exacerbations previous year**	48.0	72.0	**0.02**
**Mortality**	19.0	37.5	**< 0.001**
**BE extension** (% of patients)			
**Localised BE**	28.2	24.7	**ns**
**Bilateral BE**	48.5	57.0	**0.042**
**Diffuse BE**	23.3	18.4	**ns**

BE: bronchiectasis; ns: non-significant; sd: standard deviation

### Respiratory function data

**Fig 2:**
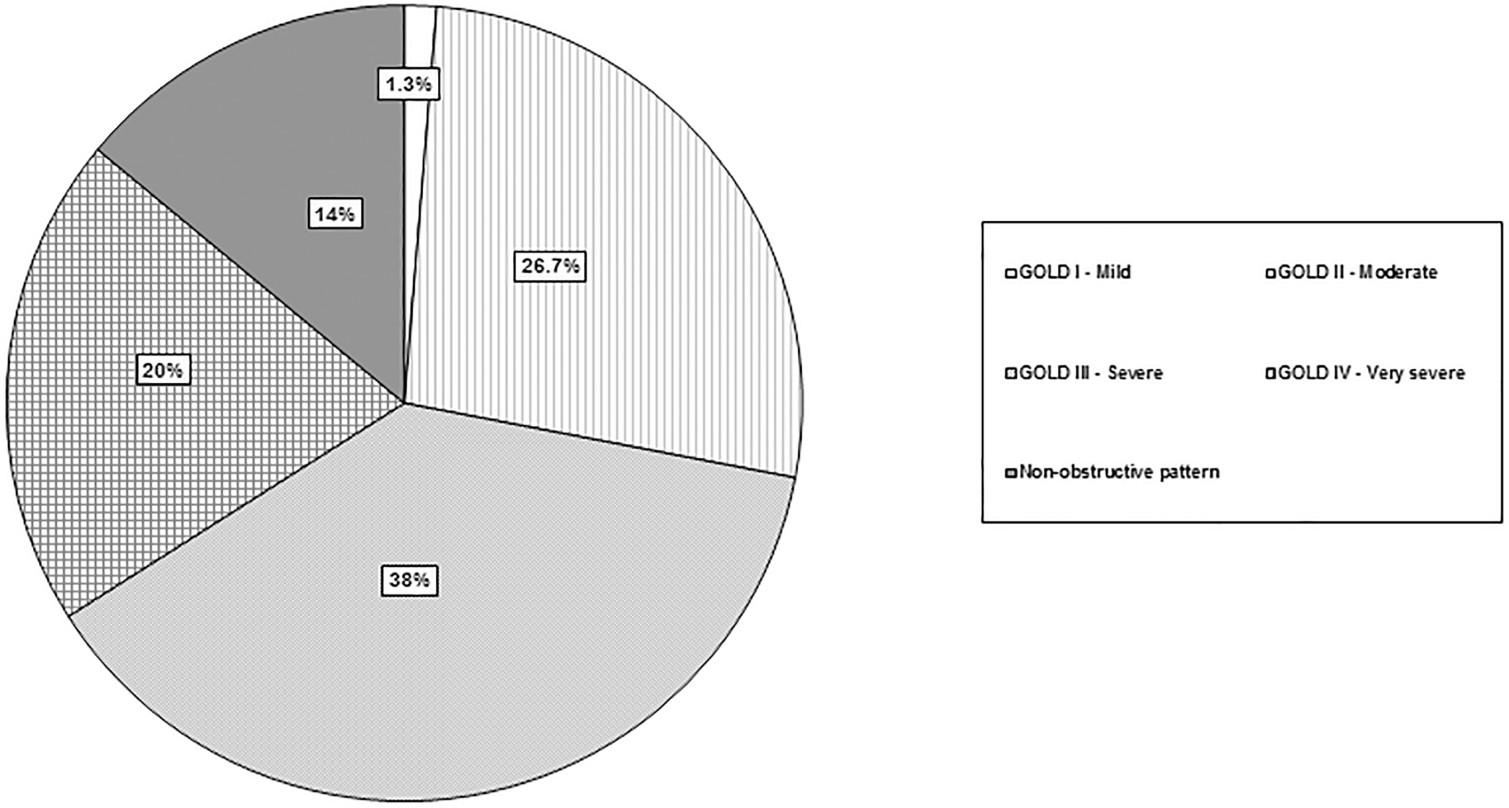
Severity of patients with bronchiectasis secondary to COPD according to GOLD staging. COPD: chronic obstructive pulmonary disease.

Table 2 shows functional comparative data between the two groups. Patients with BE and COPD had lower FVC, FEV_1_, FEV/FVC and SatO_2_ values, and most of them showed an obstructive pulmonary pattern, even though 14% presented a non-obstructive pattern (FEV_1_/FVC ≥ 0.7). A greater proportion of them had a severe or very severe functional impairment. For the data to be comparable to those of other series published in recent years, we classified the severity of COPD according to the latest GOLD criteria; 1.3% were at the stage GOLD I, 26.7% were at GOLD II, 38% were at GOLD III and 20% were at GOLD IV (Fig 2).

**Table 2:** Functional data.

	BE without COPD (n = 1,632)	BE with COPD (n = 158)	*p*
**FVC (%)** (mean ± sd)	72.8 ± 19.3	60.2 ± 16.8	**< 0.001**
**FEV**_**1**_ **(%)** (mean ± sd)	69.4 ± 23.4	45.7 ± 18.0	**< 0.001**
**FEV**_**1**_**/FVC (%)** (mean ± sd)	71.0 ± 14.5	55.3 ± 13.9	**< 0.001**
**SatO**_**2**_ **(%)** (mean ± sd)	95.2 ± 3.6	92.1 ± 4.8	**< 0.001**
**Functional severity (% of patients)**			
**Normal or mild** (FEV_1_ > 80%)	38.8	3.3	**< 0.001**
**Moderate** (FEV_1_ 50–80%)	37.3	36.7	**NS**
**Severe** (FEV_1_ 30–49%)	19.0	40.0	**< 0.001**
**Very severe** (FEV_1_ < 30%)	4.9	20.0	**< 0.001**
**Type of ventilatory disorder (% of patients)**			
**Obstructive** (FEV_1_/FVC < 0.7)	41.5	86.0	**< 0.001**
**Non-obstructive** (FEV_1_/FVC ≥ 0.7)	58.5	14.0	

FVC: forced vital capacity; FEV_1_: forced expiratory volume in 1 second; ns: non-significant; sd: standard deviation; SatO_2_: oxygen saturation.

The presence of CBC was associated with poorer respiratory function, both in patients with and without COPD. However, spirometric values were significantly lower in colonised COPD patients, compared to non-COPD colonised patients: FVC (63.3% vs. 68.1%), FEV1 (37.1% vs. 62.7%), FEV1 / FVC (48.6% vs. 62.8%) and SatO2 (91.3% vs. 94.6%) (all comparisons p<0.001).

### Microbiology data

There were no significant differences between the two groups in terms of the proportion of patients with CBC (34.9% vs. 34.2%) or in the percentage of the various isolated microorganisms. Seventeen different microorganisms were registered, the most common of which were: *P*. *aeruginosa* (PA), *H*. *influenzae* and *S*. *pneumoniae* (Table 3).

**Table 3:** Microbiological data.

	BE without COPD (n = 1,632)	BE with COPD (n = 158)	*p*
Chronic bronchial infection (% of patients)	34.9	34.2	**ns**
**Colonising microorganisms** (% of patients)			
*Pseudomonas aeruginosa*	19.8	24.7	**ns**
*Haemophilus influenzae*	12.2	8.2	**ns**
*Streptococcus pneumoniae*	5.4	3.8	**ns**
*Aspergillus spp*	3.6	2.5	**ns**
*Candida spp*	2.3	1.9	**ns**
*Staphylococcus aureus*	2.3	0.6	**ns**
*Moraxella catarrhalis*	1.3	0.6	**ns**
*Stenotrophomonas maltophilia*	0.9	1.9	**ns**
*Escherichia coli*	0.7	0	**ns**
*Klebsiella pneumoniae*	0.7	0.6	**ns**
*Acinetobacter spp*	0.6	1.3	**ns**
Atypical mycobacteria	0.6	1.3	**ns**
*Alcaligenes xylosoxidans*	0.4	1.9	**ns**
*Serratia spp*	0.4	1.3	**ns**
*Bordetella spp*	0.2	0.6	**ns**
MRSA	0.2	0.6	**ns**
*Pseudoallescheria boydii*	0.2	0	**ns**
*Scedosporium spp*	0.2	0	**ns**
*Proteus spp*	0.1	0.6	**ns**
*Nocardia spp*	0.1	0	**ns**
*Penicillium spp*	0.1	0	**ns**

MRSA: Methicillin-resistant *Staphylococcus aureus*; ns: non-significant.

**Fig 3:**
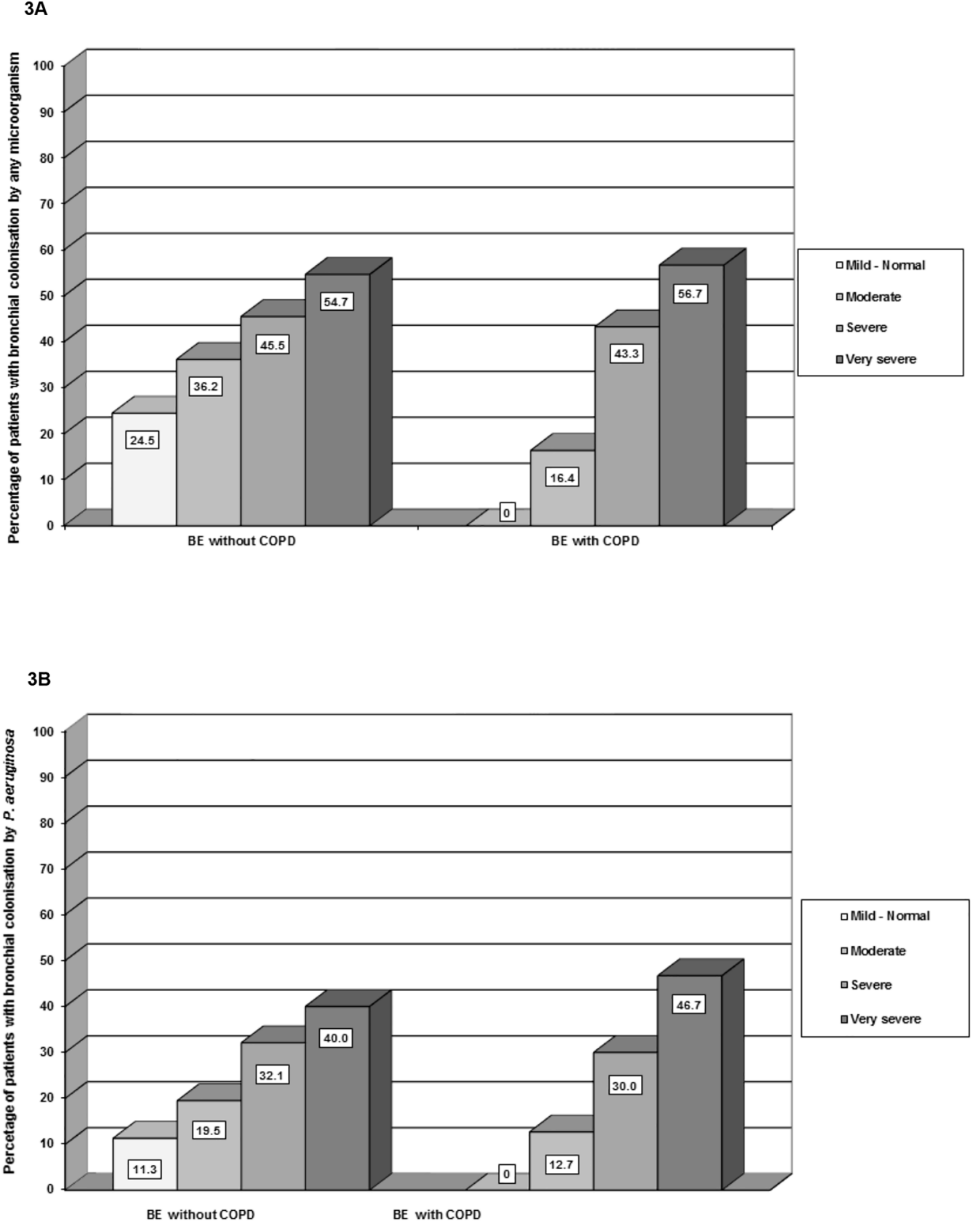
Presence of chronic bronchial colonisation. (A) Chronic bronchial colonisation by any microorganism according to functional severity group. (B) Chronic bronchial colonisation by *P*. *aeruginosa* according to functional severity group. BE: bronchiectasis: COPD: chronic obstructive pulmonary disease.

**Fig 4:**
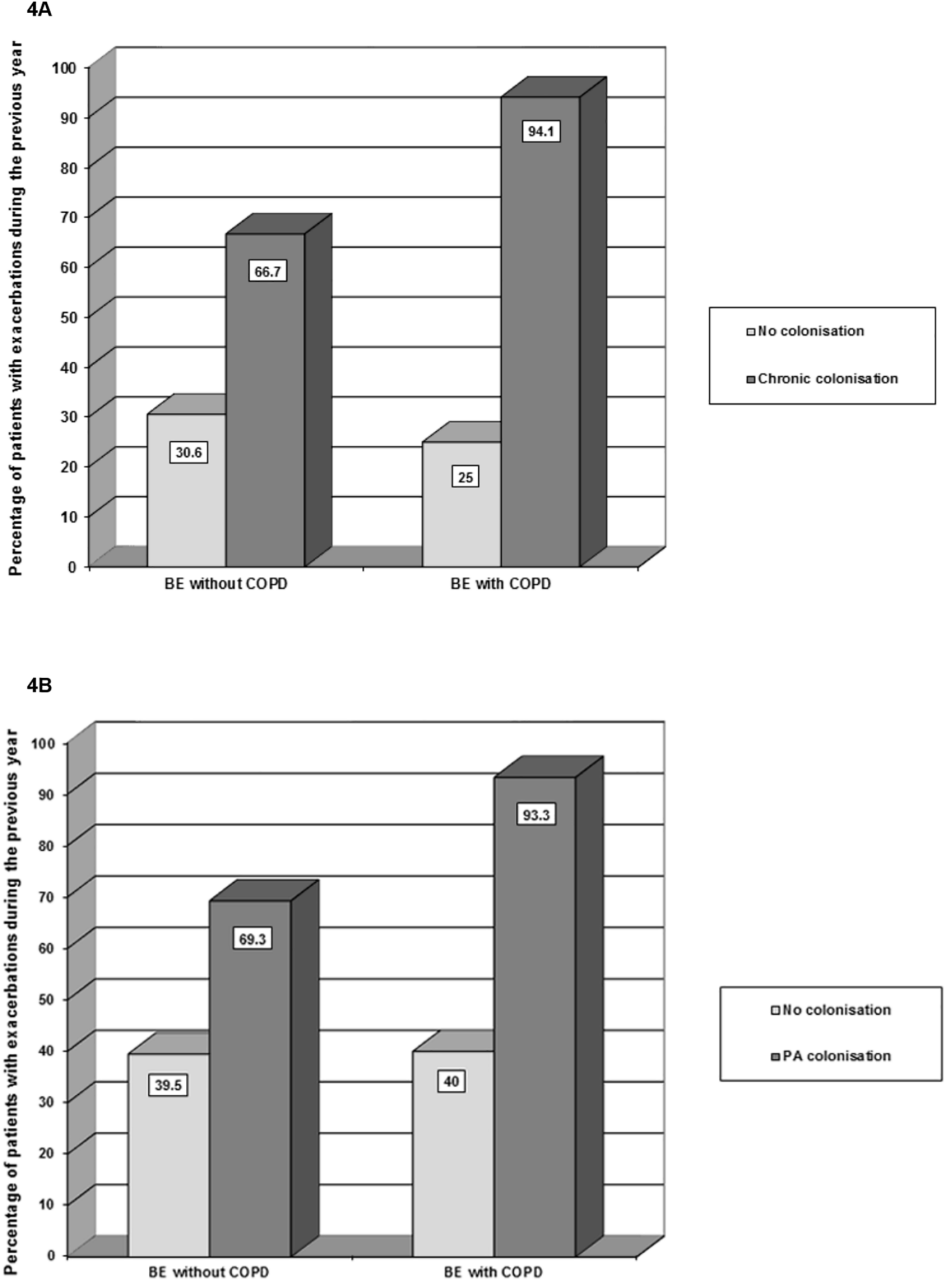
Presence of exacerbations during the previous year. (A) According to CBC by any microorganism. (B) According to CBC by *P*. *aeruginosa*. BE: bronchiectasis; CBC: chronic bronchial colonisation; COPD: chronic obstructive pulmonary disease; PA: *Pseudomonas aeruginosa*.

The proportion of patients with CBC caused by any microorganism or, more specifically, by PA increased with the functional severity impairment (in both cases p<0.001) in patients both with and without COPD, with no differences observed between the groups (Fig 3A and 3B). Furthermore, both types of CBC were significantly associated with a higher percentage of patients with exacerbations in the previous year (p<0.001 and p = 0.001, respectively). This association was more relevant in patients with COPD and CBC by any microorganism (p = 0.019) (Fig 4A and 4B).

### Treatment

Patients with BE and COPD were treated with BD and ICS and intravenous antibiotics to a larger extent, but there was no difference with non-COPD patients in the use of antibiotherapy (oral or inhaled) nor in the need for surgery (Table 4). When specifically analysing treatments in terms of CBC by PA (Table 4), we observed that there were significant differences only in ICS, which were most frequently used in COPD patients. In both groups (with/without COPD) there was a significantly greater use of ICS, oral and inhaled antibiotics in colonised patients.

**Table 4:** Treatment data according to the presence of COPD and CBC by *P*. *aeruginosa*.

	BE without COPD (n = 1,632)	BE with COPD (n = 158)	p	BE without COPD with CBC by PA (n = 323)	BE with COPD with CBC by PA (n = 39)	*p*
**Inhalers** (% of patients)						
**BD**	73.5	94.0	**<0.001**	86.7	97.2	**ns**
**Inhaled steroids**	65.3	82.7	**<0.001**	80.9	94.6	**0.039**
**Oral Antibiotherapy** (% of patients)						
**Cyclic oral antibiotics**	14.9	10.4	**ns**	30.0	26.3	**ns**
**Continuous oral antibiotics**	2.3	2.6	**ns**	7.2	5.3	**ns**
**Inhaled Antibiotherapy** (% of patients)						
**Inhaled cyclic antibiotics**	4.0	5.5	**ns**	11.9	13.5	**ns**
**Inhaled continuous antibiotics**	7.0	9.0	**ns**	25.4	24.3	**ns**
**Other treatments** (% of patients)						
**Intravenous antibiotics**	77.8	89.3	**0.004**	75.5	84.8	**ns**
**Surgery**	4.1	2.1	**ns**	5.5	5.6	**ns**

BE: bronchiectasis; CBC: chronic bronchial colonisation; COPD: chronic obstructive pulmonary disease; ns: non-significant; PA: *Pseudomonas aeruginosa*.

With respect to the use of treatment in terms of respiratory functional impairment, we only found significant differences between COPD and non-COPD patients in the use of BD (most commonly used in COPD patients with mild impairment) (p = 0.043) and cyclic oral antibiotherapy (more in non-COPD patients with moderate impairment) (p = 0.046).

### Survival analysis

**Fig 5:**
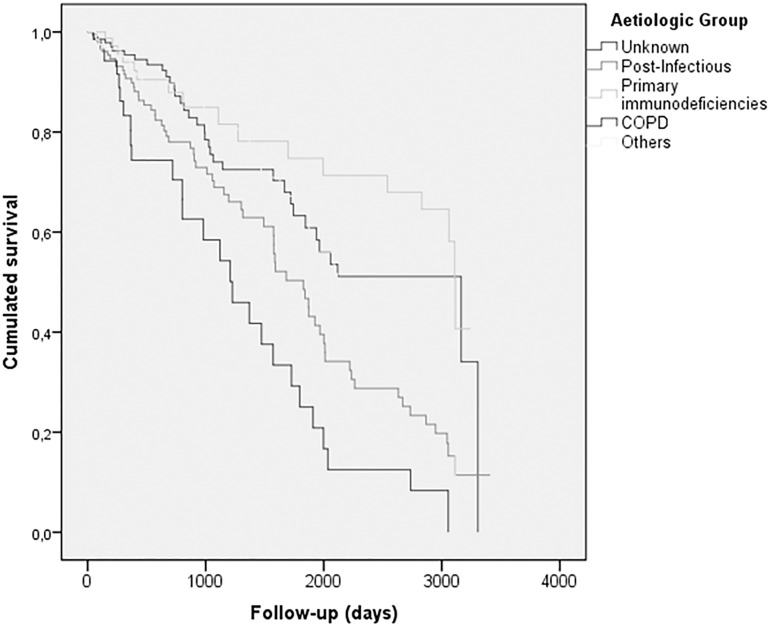
Kaplan Meier survival curve: Cumulative percentage of survivors per non-cystic fibrosis bronchiectasis aetiology. Curves compared by means of the log-rank test: COPD curve vs. other aetiologies curves (log-rank 38.800; p<0.0001).

At the time the SBHR was closed, follow-up data were available for 836 patients (46.8% of the non-COPD group and 45.6% of the COPD group), with an average follow-up time of 3.36 years. The overall proportion of deaths was 13.8%, which was significantly higher in the COPD group (37.5% vs. 19.0%; p<0.001) (Table 1). There were no significant differences in the causes of death among patients with and without COPD: respiratory (87.5% vs. 66%), cardiovascular (50% vs. 31.3%), cancer (50% vs. 56.3%) or others. When the survival between different groups was compared, patients with BE associated with COPD presented the highest mortality rate, well above post-infectious (15.3%), primary immunodeficiencies (10.1%) or unknown causes (11.1%) (Fig 5). The majority of deaths were due to respiratory causes (71.2%), although there were no significant differences between patients with or without COPD. The multivariate analysis showed that the diagnosis of COPD in a patient with BE as a primary diagnosis increased the risk of death by 1.77 compared to the rest of the patients, after adjusting for age, gender, body mass index, presence of CBC caused by PA, FEV_1_, smoking habit and BE extension (Table 5).

**Table 5:** Survival analysis: Cox regression adjusted by age, gender, BE extension, body mass index, presence of CBC caused by PA, FEV_1_ (% predicted), smoking habit and COPD diagnosis.

Variable	B	*p*	OR	IC95%
**Age**	**0.044**	**0.0001**	**1.04**	**1.02–1.07**
**COPD presence**	**0.512**	**0.001**	**1.77**	**1.18–2.76**
**FEV**_**1**_ **(% pred)**	**0.113**	**0.001**	**1.12**	**1.09–1.97**
**CBC by PA**	0.123	0.24	1.1	0.88–1.25
**Smoking habit**	0.09	0.36	1.09	0.9–1.3
**Gender**	0.134	0.12	1.06	0.91–1.34
**BE extension**	0.01	0.88	1.01	0.87–1.13
**BMI**	0.011	0.09	1.01	0.94–1.03

BE: bronchiectasis; CBC: chronic bronchial colonisation; COPD: chronic obstructive pulmonary disease; PA: *Pseudomonas aeruginosa*.

## Discussion

We analysed a homogeneous and extensive sample of patients primarily diagnosed with BE, who were subsequently diagnosed with BE related to COPD after a rigorous study. Compared to BE associated with other conditions, patients with COPD are mostly male, older, with poorer respiratory function, more frequent exacerbations and a higher mortality rate after adjusting for several confounding variables. There are no differences between them and the remaining patients as regards the proportion of CBC, colonising microorganisms or treatment regimen.

Given that this was a registry, assigning a specified aetiology to a particular patient depended on the researcher’s criteria. To avoid biases, the committee who developed and coordinated the SBHR requested that a series of recommendations be followed to diagnose and treat patients. These recommendations were later included by SEPAR in the first published clinical guidelines on diagnosing and treating this disease in 2008 [11], which were followed by the British guidelines a few years later [16]. In order to establish a diagnosis of COPD-related BE, other possible causes should have been ruled out, but patients also had to present a clinical and functional course compatible with COPD. In this respect, it was crucial that most of them were followed as outpatients on a regular basis by the participating researchers. Furthermore, the diagnosis of BE related to COPD was ruled out in patients with a history of smoking when another disease was detected in the etiological study and considered to be responsible for the BE.

Our series is the largest to have analysed the aetiology and clinical characteristics of patients with BE; we observed that in 8.8% of these, COPD was considered to be the causal factor of BE. Until recently, BE studies have included a small number of patients and also excluded those with COPD, which limited the value of the findings [17–19]. More recent studies have included a larger number of cases [20–23] but some do not include individual clinical data [23]. In these series, COPD is considered to be an aetiology of BE in a higher percentage than in ours (11–36%) [21–25]. This difference might be attributed to the fact that the SBHR’s data were collected at a time when it was not common to consider COPD as a possible cause of BE.

We found that patients with BE and COPD had more airflow obstruction and lower FEV_1_, which coincides with previous observations [5–8]. However, 14% of them presented a non-obstructive pulmonary pattern in the spirometry performed closest to the moment of inclusion in the registry. This can be attributed to technical reasons (incorrect spirometric manoeuvre), but also to factors related to COPD itself or to the presence of BE. It is known that there are patients with an obstructive pulmonary pattern who require a plethysmographic test to be diagnosed correctly [26,27], so it is not uncommon to detect a non-obstructive spirometric impairment in patients with airway diseases [27,28]. Moreover, a restrictive element is a feature of previous functional research into BE [28,29], and no satisfactory explanation has been advanced for this finding. Since our study was cross-sectional, the spirometry values introduced at the time of inclusion in the registry were maintained, even in the 21 COPD patients with no obstructive pattern. However, by contacting the responsible researchers we ensured that these patients had COPD-compatible clinical/epidemiological characteristics and spirometries with an obstructive pattern during their clinical course, and that other causes of BE had been ruled out.

The frequency of CBC and the proportion of colonising microorganisms were similar in patients with and without COPD (34.9% vs. 34.2%), and CBC by PA was associated with increased functional impairment and increased frequency of exacerbations. Given that we were comparing two groups of patients with BE, no differences were expected, since the association between CBC, low FEV_1_ and presence of exacerbations [30,31] is already known.

It is noteworthy that a large proportion of patients received ICS, particularly those with CBC. Subsequent to warnings of a higher risk of pneumonia in COPD patients being treated with ICS [32], there has been a debate on the advisability of restricting its use, especially when CBC is present. Recent studies have revealed an increase in dose-dependent bacterial load in patients who receive ICS [33], which could be due to a decrease in local immune defences that would favour bacterial growth. Until there is more available evidence, use of ICS in BE should be limited to patients with bronchial hyper-responsiveness, as recommended by the guidelines [11,16].

CBC by PA in patients with BE of any aetiology is associated with a poorer clinical evolution [31,34,35], which leads to an attempt to reduce bacterial load with long-term antibiotherapy. Participants in the SBHR coincided in their use of oral and inhaled antibiotics to treat CBC in patients with BE related to COPD, even though these treatment regimens are not included in the COPD treatment guidelines [36]. The effectiveness of long-term macrolides has been evaluated in COPD and BE with favourable results [37,38] but the use of other oral antibiotics to avoid exacerbations in COPD has had highly variable results: consequently, the systematic use of such drugs is not recommended at this time [39,40]. Only two studies have evaluated the use of inhaled antibiotics—tobramycin and levofloxacin—in a few COPD patients, with inconclusive results [41,42]. Despite the lack of scientific evidence, it is increasingly common in everyday clinical practice to treat CBC by PA in patients with BE with inhaled antibiotics, regardless of their aetiology.

The survival analysis of the 836 patients in whom follow-up data were available showed a significantly increased mortality in COPD patients (26.2%). The presence of COPD in patients who had been initially diagnosed with BE increased the risk of death by 1.87 times, in line with previous observations [9,43]. Goemmine et al [9] analysed survival in a series of 245 patients, and also observed that patients with BE related to COPD had a higher mortality (55%), with the risk of death increased 2.12 times. The difference with our study as regards mortality may be due to the longer follow-up period (5.18 years), but also to the higher average age of the patients included in their study. Despite having a sample that was three times larger, we observed, like Goemmine et al, that patients with BE and COPD had a significantly higher mortality than those of other BE aetiologies. This confirms the impression that these patients form a special phenotype, different from COPD without BE, but also different from the rest of non-CF BE.

Our study has several strengths, since we had a wide and homogeneous sample of patients monitored by pulmonologists at specialised outpatient clinics for BE in different autonomous communities in Spain, with data being collected systematically. Furthermore, we have studied the characteristics of COPD patients in a BE patient cohort, in contrast to most of the studies in this respect, which assess BE patients in a COPD patient cohort.

Our study has also some limitations, however. One of them is that the diagnosis of BE related to COPD depended on the clinical judgement of the researcher involved, which could favour a selection bias. Nevertheless, in order to assign a specific aetiology, researchers were requested to reasonably rule out every other cause of BE, following a study protocol that led to an etiological diagnosis in 72.2% of patients. Furthermore, in cases where doubts about the diagnosis of COPD might arise, as in patients with no spirometric obstruction, the corresponding researcher was contacted to minimise the possibility of a misdiagnosis. We only considered BE to be associated with COPD after confirmation of spirometric obstruction (and exclusion of other causes of BE) in those patients whose physicians thought COPD was a key factor due to smoking and medical history, or due to the presence of emphysema in CT (data not recorded in the registry). Another limitation is the absence of some relevant information due to the simplicity of the data collection process: active principles and doses of the medication used; confirmation of the quality of the spirometries; and data allowing the calculation of the FACED [20] and BSI [44] severity scores. Finally, mortality data were available in only 836 patients, but they were similarly distributed between patients with and without COPD, so we consider the comparative survival analysis between the two groups to be valid.

In conclusion, patients with BE related to COPD show the same type of microbiological characteristics and receive the same treatment regimens as patients with BE from other aetiologies, with a surprisingly high use of ICS. These patients, monitored by pulmonologists specialised in BE, follow a treatment aimed at controlling CBC by PA with long-term oral and inhaled antibiotics, despite the fact that current COPD treatment guidelines do not envisage this type of therapies. Their mortality is notably higher than that of the remaining patients with non-CF BE. We believe these results would justify carrying out prospective studies aimed at assessing the tolerance and efficiency of these treatments in COPD patients.

## Supporting information

S1 File List of aetiologies. Complete list of individual and grouped etiologies reported by researchers.
(DOCX)
