# Supplementary material for: Clinical impact of chronic obstructive pulmonary disease on non-cystic fibrosis bronchiectasis. A study on 1,790 patients from the Spanish Bronchiectasis Historical Registry
Source: PLoS One. 2017 May 18;12(5):e0177931. doi: 10.1371/journal.pone.0177931 (PMC5436841; doi:10.1371/journal.pone.0177931)
Supplement: S1 File — Complete list of individual and grouped etiologies reported by researchers. (DOCX) [file pone.0177931.s001.docx]

|  | **Cause** | % |
| --- | --- | --- |
|  | **Unknown** | 27,7 |
|  | **Post-tuberculous** | 21,2 |
|  | **Non-tuberculous infection** | 13,0 |
|  | *Childhood infections (9,8)* |  |
|  | *Necrotizing pneumonia (3,0)* |  |
|  | *Non-tuberculous mycobacteria (,2)* |  |
|  | **Primary Immunodeficiencies** | 10,7 |
|  | *Immunoglobulin deficiency (9,2)* |  |
|  | *Common Variable Immunodeficiency (1,1)* |  |
|  | *Other immunodeficiencies (,3)* |  |
|  | *Complement deficiency (,1)* |  |
|  | **COPD** | 8,8 |
|  | **Asthma** | 6,1 |
|  | **Ciliary disease** | 3,3 |
|  | **Connective tissue disease** | 1,6 |
|  | **Aspiration** | 1,1 |
|  | **ABPA** | 1,0 |
|  | **GERD** | ,7 |
|  | **Tracheobronchial malformations** | ,6 |
|  | **α1-antitrypsin deficiency** | ,5 |
|  | **Localized bronchial obstruction** | ,5 |
|  | **Gas inhalation** | ,3 |
|  | **Young’s syndrome** | ,3 |
|  | **Inflammatory bowel disease** | ,3 |
|  | **Pulmonary embolism - Pulmonary infarction** | ,3 |
|  | **Yellow nail syndrome** | ,2 |
|  | **HIV** | ,2 |
|  | **Post-transplant** | ,2 |
|  | **Drug inhalation** | ,2 |
|  | **Purulent rhinosinusitis** | ,2 |
|  | **Swyer-James-Mc Leod syndrome** | ,2 |
|  | **Vasculitis** | ,1 |
|  | **Panbronchiolitis** | ,1 |
|  | **Hydatid cyst** | ,1 |
|  | **Pulmonary fibrosis** | ,1 |

**Etiologies**

**Pooled etiologies**

| **Cause** | n | % |
| --- | --- | --- |
| **Unknown** | 496 | 27,7 |
| **Post-tuberculous** | 380 | 21,2 |
| **Non-tuberculous infection** | 233 | 13,0 |
| **Primary Immunodeficiencies** | 192 | 10,7 |
| **COPD** | 158 | 8,8 |
| **Miscellaneous** | 143 | 8,0 |
| **Asthma + ABPA** | 128 | 7,2 |
| **Ciliary disease** | 60 | 3,4 |
